# L-leucine and SPNS1 coordinately ameliorate dysfunction of autophagy in mouse and human Niemann-Pick type C disease

**DOI:** 10.1038/s41598-017-15305-9

**Published:** 2017-11-21

**Authors:** Hiroko Yanagisawa, Tomohiro Ishii, Kentaro Endo, Emiko Kawakami, Kazuaki Nagao, Toshiyuki Miyashita, Keiko Akiyama, Kazuhiko Watabe, Masaaki Komatsu, Daisuke Yamamoto, Yoshikatsu Eto

**Affiliations:** 1 Advanced Clinical Research Center, Institute for Neurological Disorders, Kawasaki, Japan; 2 Division of Pharmacology, Faculty of Pharmacy, Keio University, Tokyo, Japan; 3 Center for Basic Technology Research, Tokyo Metropolitan Institute of Medical Science, Tokyo, Japan; 4 Department of Medical Technology, Faculty of Health Sciences, Kyorin University, Tokyo, Japan; 5 Department of Biochemistry, Graduate School of Medical and Dental Sciences, Niigata University, Niigata, Japan; 6 Division of Neurogenetics, Graduate School of Life Science, Tohoku University, Sendai, Japan; 7 Department of Molecular Genetics, Kitasato University Graduate School of Medical Sciences, Sagamihara, Japan

## Abstract

Lysosomal storage disorders are characterized by progressive accumulation of undigested macromolecules within the cell due to lysosomal dysfunction. 573C10 is a Schwann cell line derived from a mouse model of Niemann-Pick type C disease-1, NPC (−/−). Under serum-starved conditions, NPC (−/−) cells manifested impaired autophagy accompanied by an increase in the amount of p62 and lysosome enlargement. Addition of L-leucine to serum-starved NPC (−/−) cells ameliorated the enlargement of lysosomes and the p62 accumulation. Similar autophagy defects were observed in NPC (−/−) cells even without serum starvation upon the knockdown of Spinster-like 1 (SPNS1), a putative transporter protein thought to function in lysosomal recycling. Conversely, *SPNS1* overexpression impeded the enlargement of lysosomes, p62 accumulation and mislocalization of the phosphorylated form of the mechanistic Target of rapamycin in NPC (−/−) cells. In addition, we found a reduction in endogenous *SPNS1* expression in fibroblasts derived from NPC-1 patients compared with normal fibroblasts. We propose that SPNS1-dependent L-leucine export across the lysosomal membrane is a key step for triggering autophagy, and that this mechanism is impaired in NPC-1.

## Introduction

Autophagy is a mechanism for generating nutrients for survival under starvation conditions. A key player in autophagy is the autophagosome, an organelle delineated from the cytoplasm by a lipid bilayer, which fuses with the lysosome to be digested, yielding nutrients for metabolism during fast. Consequently, autophagic dysfunction is involved in various disorders, namely, Parkinson’s disease, Alzheimer’s disease, and a variety of polyglutamine diseases and cancer^1^. Lesions in biochemical pathways for the control of autophagy have been reported in several types of lysosomal storage disorder (LSD)^2^. Niemann-Pick type C disease (NPC) is a representative of LSDs, arising from lipid trafficking defects typically caused by mutations in the *NPC1* ^3^ or *NPC2* ^4^ gene. NPC symptoms include ataxia, cataplexy, cognitive decline, dystonia and vertical supranuclear gaze palsy. At the subcellular level, NPC is characterized by impairments in the clearance of autophagosomes, as a consequence of an inhibition of lysosomal protease activity by stored lipids^5^. Studies on disease models, including *npc1* (−/−) mice, have provided clues for understanding how defects in autophagy contribute to pathogenesis^6^.

On the other hand, knockdown of the *Spinster-like 1* (*SPNS1*) gene induces the accumulation of enlarged lysosomes in fibroblast-derived NRK cells, providing another model for LSD^7^. *spinster* (*spin*) is a gene first characterized in *Drosophila melanogaster*, originally defined by a mutation in which the females exhibited a strong rejection behavior toward courting males^8,9^. The *spin* gene encodes a putative lysosomal efflux permease belonging to the major facilitator superfamily^7,10–12^. In *spin* mutant *Drosophila*, programmed cell death is impaired in germline nurse cells in the ovary as well as neurons and glia, which accumulate lipofuscin-like materials, implying that *spin* is involved in the regulation of lysosomal turnover^12^. When overexpressed in HEK293 cells, the human *spin* homolog *SPNS1* (*HSpin1*) induced cell death, which was accompanied by increases in autophagic vacuoles and the production of the mature form of cathepsin D^13^ These seminal works that implicate Spin/SPNS1 in autophagy prompted us to examine the possible involvement of SPNS1 in LSD pathology using a Schwann cell line 573C10 derived from the NPC1 model mouse^14^, which we refer to as NPC (−/−) cells hereafter. In this work, we show that the addition of L-leucine to the medium for NPC (−/−) culture or the overexpression of *SPNS1* in NPC (−/−) cells suppresses both the enlargement of lysosomes and the p62 accumulation induced by serum starvation.

## Results and Discussion

### NPC (−/−) cells manifest autophagy defects under serum starvation

**Figure 1:**
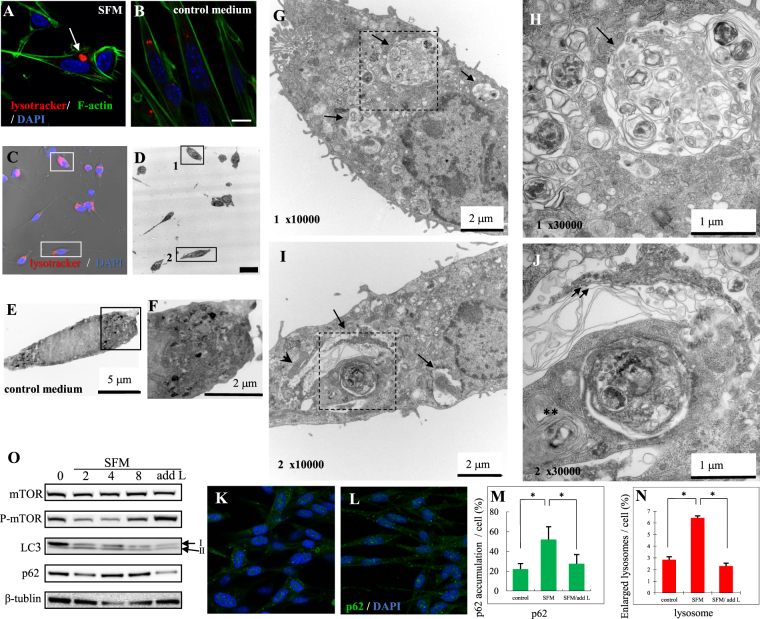
L-leucine mitigates dysfunction of autophagy in NPC (−/−) cells. (**A**,**B**) Serum starvation induces an enlargement of lysosomes. NPC (−/−) cells were triply stained with lysotracker to visualize lysosomes (red), fluorescent phalloidin to label F-actin (green) and DAPI to mark DNA (blue). When kept in a serum-free medium (SFM) for 8 h, NPC (−/−) cells developed a larger number of enlarged autolysosomes (arrows in panel A) than did the cells maintained in a control medium (control; **B**). Scale bar; 10 µm. (**C**,**D**) EM observations reveal ultrastructural abnormalities of intracellular organelles in NPC (−/−) cells exposed to serum starvation. The identical field of NPC (−/−) cells kept in SFM was observed by fluorescence microscopy in panel (C) and by electron microscopy in panel (D). Scale bar: 20 µm. The cells indicated by box-1 and box-2 in (**D**) are observed at higher magnifications in panels (**G**,**H**) for box-1 and panels (**I**,**J**) for box-2. Scale bar: 2 µm (**G**,**I**), 1 µm (**H**,**J**). (**E**,**F**) An NPC (−/−) cell kept in a normal medium. The boxed region in (**E**) is enlarged in (**F**). (**G**,**J**) Higher magnification images of serum-starved NPC (−/−) cells. Autolysosomes (arrow), mitochondria (arrowhead), lamellar inclusions (asterisks) and skein-like inclusions (double arrow) are highlighted. (**K**–**N**) L-leucine supplementation rescues autophagy defects in serum-starved NPC (−/−) cells. Images of serum-starved NPC (−/−) cells stained for p62 (green) and DAPI (blue) are shown before (**K**) and after (**L**) L-leucine administration. The relative areas containing p62 immunoreactive materials in a cell (%) were analyzed by the imaging program MetaMorph (**M**). The areas containing lysotracker signals in a cell (%) are shown in (**N**). Cells were analyzed in 13 (p62) or 25 (lysosome) fields chosen at random for each condition (Dunnet post-test *p < 0.05). NPC (−/−) cells were serum-starved for 8 h, followed by a 16-h incubation with 10 mM L-leucine dissolved in a serum-free DMEM. SFM: cells in a serum free medium; SFM/add L: cells treated with L-leucine. (**O**) Western blot analysis of the indicated proteins in NPC (−/−) cells sampled at different time points (in h) before (0), and after (2–8) the replacement of a normal culture medium with a serum-free medium (SFM) and 16 h after the treatment with L-leucine (dissolved in a serum-free DMEM).

To address whether serum starvation affects the structure of lysosomes of NPC (−/−) cells, we employed a lysotracker to label the lysosomes in these cells, which were counterstained with phalloidin for F-actin and DAPI for DNA (Fig. 1A). Upon serum starvation for 8 h, NPC (−/−) cells developed enlarged lysosomes (SFM: Fig. 1A and N), which were 2~3 times more abundant than in a control culture (Control: Fig. 1B and N). After observing under the fluorescence microscopy, the same cell was subjected to the observation under the electron microscopy (EM). The EM observation revealed that serum-starved NPC (−/−) cells have enlarged vacuoles, which were accompanied with lamellar inclusions and abnormal membrane structures (Fig. 1G–J). It is likely that the enlarged vacuoles in the EM images correspond to the organelles marked with lysotracker in fluorescent microscopic images, because they have features typical of autolysosomes^15^: these EM structures were delineated from the cytoplasm by a single membrane and contained mitochondria (Fig. 1I). We suggest that autolysosomes of NPC (−/−) cells transform into enlarged vacuoles upon serum starvation, reflecting defective autophagy.

### L-leucine mitigates autophagy defects in NPC (−/−) cells

SPNS1 is structurally similar to the vacuolar effluxer Atg22, which transports, from vacuoles to cytoplasm, autophagic degradation products, including L-leucine^16^, an important regulator of autophagy. We therefore examined a possible effect of L-leucine addition to the culture medium on autophagy dysfunction in NPC (−/−) cells. Serum starvation-induced p62 accumulation in these cells was reduced by the addition of L-leucine (Fig. 1K,L). To evaluate quantitatively the L-leucine effect, we measured the areas with p62 accumulation (small foci with a radius of less than 0.5 μm were not included), and the areas occupied by enlarged lysosomes (small foci with a radius of less than 1.0 m were not included) using an imaging software package, MetaMorph (Molecular Devices Corp.). The quantitative analysis showed that L-leucine supplementation prevents the NPC (−/−) cells from accumulating p62 and enlarged lysosomes (Fig. 1M,N). In keeping with this cytochemical observation, Western blot analysis (WB) revealed a reduced expression of p62 after L-leucine administration (Figs 1O, S1 and S9). This phenomenon implies that L-leucine is a restorative agent for autophagy flux defects. There are reports that manipulations of the L-leucine level may modulate autophagy; for example, leucine limitation has been shown to induce autophagy in C2C12 myotubes^17^. In contrast, a reduction in muscle mass was suppressed by leucine supplementation in rats fed a protein-fee diet^18^. In human myotubes, leucine activates mechanistic Target of rapamycin (mTOR) signaling^19^, a key transduction cascade for the regulation of autophagy^20^.

To determine which component in the autophagic machinery is impaired in NPC (−/−) cells, we examined whether mTOR is inactivated by dephosphorylation, because this step is known to be essential for starvation-induced autophagy^21^. We found no sign of mTOR dephosphorylation upon a starvation challenge in NPC (−/−) cells (Figs 1O, S1, S8 and S9). We suggest that a failure in dephosphorylation of mTOR is, at least in part, responsible for the impaired autophagy in serum-starved NPC (−/−) cells.

### Serum starvation induces mislocalization of phosphorylated-mTOR in NPC (−/−)

**Figure 2:**
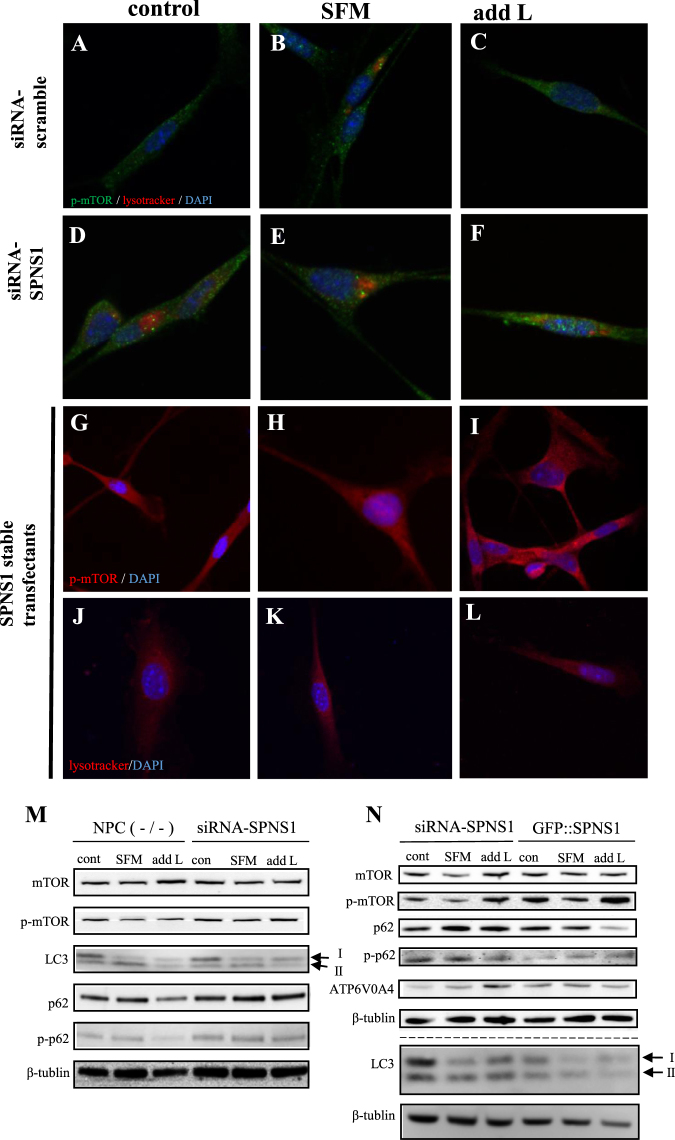
Effects of *SPNS1* knockdown and overexpression on autophagy defects of serum-deprived NPC (−/−) cells with or without L-leucine supplementation. (**A**–**F**) Localization of phosphorylated mTOR (p-mTOR) (green) and lysotracker (red) signals in NPC (−/−) cells without (**A**–**C**) or with (**D**–**F**) *SPNS1* knockdown kept in a normal culture medium (control: **A**,**D**), serum-free-medium (SFM: **B**,**E**) or L-leucine supplemented serum-free medium (add L: **C**,**F**). (**G**–**I**) Localization of phosphorylated mTOR (p-mTOR) (red) in NPC (−/−) cells with *SPNS1* stable expression kept in a normal culture medium (control: **G**), serum-free-medium (SFM: **H**) or L-leucine supplemented serum-free medium (add L: **I**). (**J**–**L**) Lysotracker (red) signals in NPC (−/−) cells with *SPNS1* stable expression kept in a normal culture medium (control: **J**), serum-free-medium (SFM: **K**) or L-leucine supplemented serum-free medium (add L: **L**). (**M**) Western blot analysis of the indicated proteins in NPC (−/−) cells without (left) or with (right) *SPNS1* knockdown (via *siRNA-SPNS1*). Cells were kept in a normal culture medium (con), serum-free medium (SFM) or serum-free medium supplemented with L-leucine (add L). The arrows indicate two forms of LC3 protein (LC3I and LC3II). (**N**) Western blot analysis of the indicated proteins in NPC (−/−) cells with *SPNS1* knockdown (siRNA-SPNS1) or NPC (−/−) cells with stable *SPNS1* expression (GFPSPNS1). Cells were kept in a normal culture medium (con), serum-free medium (SFM) or serum-free medium supplemented with L-leucine (add L). Arrows indicate two forms of LC3 protein (LC3I and LC3II).

It has been shown that starvation induces clustering of lysosomes at the perinuclear region of a cell accompanied by inactivation of the mTOR complex 1 (mTORC1), whereas nutrient replenishment results in the scattering of lysosomes around the cell periphery with concomitant activation of mTORC1 by nutrients^22^. We therefore checked the localization of phosphorylated mTOR in NPC (−/−) cells before and after serum starvation. In a control medium, phosphorylated mTOR was located in peripheral lysosomes (Fig. 2A), whereas, during starvation, phosphorylated mTOR was preferentially localized in the perinuclear area, in association with closed enlarged lysosomes (Fig. 2B). Moreover, addition of L-leucine to the nutrient-deprived NPC (−/−) culture led to altered positioning of phosphorylated mTOR and lysosomes: they were enriched in the periphery rather than in the perinuclear region (Fig. 2C). In this context, it is noteworthy that Leucyl-tRNA synthetase (LRS) plays a critical rule in amino acid-induced mTORC1 activation by sensing the intracellular leucine concentration^23^. Our present observations, together with other published results^24^, are compatible with the hypothesis that L-leucine restores arrested autophagy by relocating lysosomes at the cell periphery so that mTORC1 can be phosphorylated and thus activated by extracellular nutrients.

In *Drosophila*, the putative lysosomal efflux permease Spin has been implicated in mTOR activation upon starvation^7,25^. To test for the possible involvement of SPNS1 in the observed rescue by L-leucine, we examined the effect of *SPNS1*-knockdown in NPC (−/−) cells on the subcellular localization of phosphorylated mTOR, since this likely represents a key step for the L-leucine-mediated rescue of autophagy defects in these cells. In NPC (−/−) cells with *SPNS1*-knockdown that were maintained in a normal medium, phosphorylated mTOR was preferentially localized to closed enlarged lysosomes in the perinuclear area (Fig. 2D). L-leucine addition to the nutrient-deprived NPC (−/−) culture failed to relocate lysosomes and associated phosphorylated mTOR to the cell periphery (Fig. 2F). In addition, serum-starved NPC (−/−) cells with *SPNS1* knockdown were unable to block an increase in total and phosphorylated p62 upon L-leucine administration, whereas, in NPC (−/−) cells with functional *SPNS1*, the same treatment effectively mitigated p62 elevation (Figs 2M, S2, S12 and S13). In contrast to the NPC (−/−) cells with *SPNS1* knockdown, cells with overexpression of *GFP*-tagged *SPNS1* (*GFP::SPNS1*) exhibited a lower level of total and phosphorylated p62 than control NPC (−/−) cells did (Figs 2N, S3, S15 and S16). Here we measured phosphorylated p62 as an indicator of cell damage, based on a report that the assembly of p62 on ubiquitinated aggregates stimulates p62 phosphorylation, followed by activation of nuclear factor erythroid 2-related factor (Nrf2)^26^. Thus, our results suggest that cell damage was reduced by *GFP::SPNS1*. We also noted that the expression level of phosphorylated mTOR was higher in cells with *GFP::SPNS1* than in cells in which *SPNS1* was knocked down by siRNA (Figs 2N, S3, S15 and S16). To ascertain that *SPNS1* was successfully overexpressed or knocked down, we performed qPCR analysis for *SPNS1* mRNA in these cells. We detected an increase and decrease of *SPNS1* mRNA expression by overexpression and knockdown of *SPNS1*, respectively (Fig. S4).

We conclude that SPNS1 and L-leucine coordinately rescue autophagy defects in NPC (−/−) cells, although the underlying mechanism remains to be explored. Notably, SPNS1 has been implicated in autophagic lysosome reformation^7^^,^^25^ (ALR), which is a prerequisite for the recurrent autophagosome-lysosome fusions. In cells with overexpression of *SPNS1*, lysosomes and phosphorylated mTOR were diffusely distributed in the cytoplasm (Fig. 2G–L). This suggests that SPNS1 might regulate luminal solute compositions, thereby altering the subcellular distribution of lysosomes. Taking all these observations into account, we postulate that SPNS1 contributes, at least in part, to L-leucine flux across the lysosome membrane via ALR and thereby contributes to the regulation of intracellular L-leucine concentrations.

### Fibroblasts from NPC1 patients are defective in autophagy

**Figure 3:**
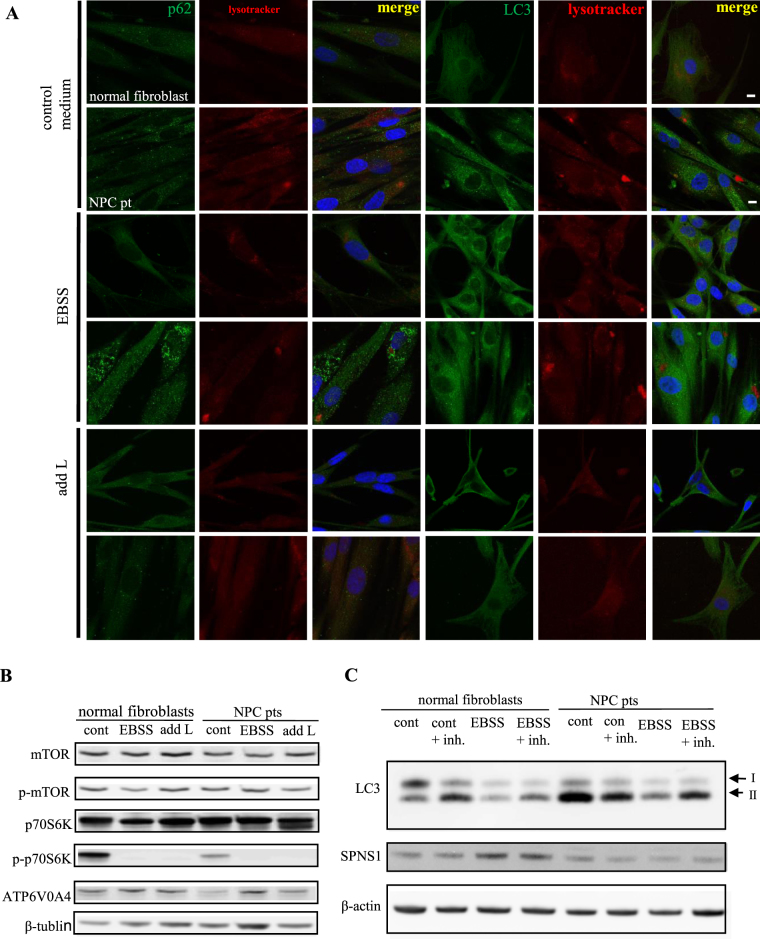
Dysfunction of autophagy in fibroblasts derived from NPC1 patients. (**A**) Autophagic hypofunction in NPC patient cells is rescued by adding L-leucine. NPC-patient-derived or normal fibroblasts kept in a normal culture medium (control medium), serum-free Earl’s balanced salt solution (EBSS), or EBSS supplemented with L-leucine (add L) were stained with lysotracker, DNA (DAPI) and p62 or LC3. (**B**) Western blot analysis of the indicated proteins in NPC1 patient cells kept in a normal culture medium (con), EBSS (EBSS) or L-leucine supplemented EBSS (add L). (**C**) Western blot analysis of LC3 and SPNS1 in fibroblasts from healthy subjects (normal fibroblasts) or from NPC1 patients (NPC pts) kept in a normal culture medium (con), a normal culture medium that contained protease inhibitors, pepstatin A and E64d (con + inh.), EBSS (EBSS), or EBSS that contained the inhibitors (EBSS + inh.). Arrows indicate two forms of LC3 protein (LC3I and LC3II). β-actin served as a loading control.

We then examined fibroblasts from NPC1 patients for dynamics in the molecular markers of autophagy to ascertain that our findings in mouse NPC (−/−) cells explain aspects of pathology in human NPC. We found that serum/amino acid-starvation (EBSS) induces an accumulation of enlarged autolysosomes and of p62 in fibroblasts from NPC1 patients but not in normal fibroblasts (Figs 3A and S5). Moreover, a striking difference between the normal and patient fibroblasts was found in phosphorylated p70S6K (p-p70S6K), a downstream target of phosphorylated mTOR (p-mTOR); the fibroblasts from patients exhibited a very low level of p-p70S6K expression compared with normal fibroblasts (Figs 3B, S6, S19 and S20). This result was in good agreement with a report^27^ that the ratio of p-S6K relative to total S6K was decreased in acid alpha-glucosidase KO cells, a Pompe disease model, in which mTORC1 activity is reduced. Next, we checked the expression of LC3, a protein involved in formation of the autophagosomal membrane, in normal and NPC patient-derived fibroblasts. In NPC patient fibroblasts, LC3 immunoreactivity had a punctuate appearance and was distributed in the perinuclear area (Fig. 3A) even without serum/amino acid starvation, in contrast to normal fibroblasts, where LC3 was distributed in the normally localized periphery and, upon starvation, accumulated in the perinuclear area (Figs 3A and S5). We further attempted to estimate autophagic fluxes using LC3-turnover assays^28^. The rate of degradation of LC3II was estimated by comparing two samples respectively prepared in the presence and absence of lysosomal inhibitors. In a normal culture medium, fibroblasts from healthy subjects increased their retention of LC3II in response to lysosomal inhibitors, whereas fibroblasts from NPC1 patients revealed an excess amount of LC3II even in the absence of inhibitors and did not show an additional increase in the amount of LC3II in response to inhibitors (Figs 3C, S7 and S22). Intriguingly, the expression level of SPNS1 was lower in fibroblasts from NPC patients than in normal fibroblasts (Figs 3C, S7 and S23). It appears that the level of LC3II expression is inversely correlated with that of SPNS1 (Figs 3C, S7, S22 and S23). Based on these results, we conclude that autophagy flux is reduced in NPC patient cells, and this reduction is at least partly ascribable to a reduction in SPNS1 expression in these cells.

### Possible roles of SPNS1 in autophagy

In vertebrates, there exist three members of SPNS, i.e., SPNS1, SPNS2 and SPNS3, and their functions have not been fully unraveled. Recently, *Spns2*-deficient mice were found to have a significantly reduced number of metastatic foci compared with their wild type counterparts^29^. Studies on zebrafish mutants with the cardia bifida (two hearts) phenotype led to identification of the zebrafish homolog of *Spns2*, which was shown to encode a sphigosine-1-phosphate (S1P) transporter with a role in S1P secretion^30,31^. Another study in zebrafish revealed that developmental senescence due to *Spns1* deficiency can be suppressed by a concurrent disruption of the vacuolar-type H^+^-ATPase (v-ATPase) subunit gene, *atp6v0ca* ^32^, implying that loss of *Spns1* resulted in an elevated v-ATPase activity, which may have partly contributed to the *Spns1*-loss phenotype. However, we also obtained a result in conflict with this report: *SPNS1* overexpression increased, rather than decreased, the amount of ATP6V0A4, though the effect was statistically non-significant (Figs 2N, S3 and S15). Conversely, in NPC-patient fibroblasts, the amount of ATP6V0A4 was significantly decreased (Fig. 3B, S6 and S19). It might be that SPNS1 promotes v-ATPase activities, thereby stimulating efflux of L-leucine from the lysosome lumen to cytosol, a key step for the regulation of autophagy fluxes. The acidic lysosomal lumen (pH 4–5) is of critical importance for the activities of most hydrolytic enzymes involved in autophagy^33^. Lysosomal acidification is dependent on the activity of the v-ATPase proton pump^34^ as well as on ClC-7, a Cl^−^/H^+^ exchange^35^. These considerations lead us to speculate that SPNS1 operates to maintain the acidity of the intra-lumen compartment. Although this hypothesis awaits rigorous experimental testing, our present findings implicate the importance of SPNS1 in lysosomal homeostasis, and thus suggest a novel target for future LSD therapy.

## Materials and Methods

### Antibodies

For Western blotting and immunofluorescence, the following primary antibodies were used: rabbit anti-LC3 [PM036] (1:100 for immunofluorescence; 1:500 for Western blot), rabbit anti-phospho-p62 [PM074] (Ser351) (1:500) from Medical & Biological Laboratories (MBL); rabbit anti-mTOR [#2972] (1:1000), rabbit anti-phospho-mTOR (Ser2448) [#2971] (1:100 for immunofluorescence; 1:1000 for Western blot), rabbit anti-p70S6 kinase [#2708] (1:1000), and rabbit anti-phospho-p70S6 kinase (Thr389) [#2708] (1:1000) from Cell Signaling; guinea pig anti-p62 [P0G-GP62-C] (1:1000) from Progen; rabbit anti-ATP6V0A4 [ab97440] (1:1000) from AbCam; mouse anti-β-tubulin [T4029] (1:1000) and mouse anti-β-actin [A5316] (1:1000) from SIGMA; and rabbit Spin1/SPNS1 (1:100) from original antibodies^12^.

### Reagents

The chemicals used were pepstatin A (SIGMA), E-64-d (PEPTDE), and L-leucine (SIGMA).

### Cell culture

57310 C was cultured in a humidified 5% CO_2_, 37 °C incubator in Dulbecco’s modified Eagle’s medium (DMEM; SIGMA) supplemented with 5% fetal bovine serum (FBS; Life Technologies/Gibco), 2 mM L-glutamine (Life Technologies/Gibco), and 100U penicillin/streptomycin (SIGMA).

Human fibroblasts were cultured in a humidified 5% CO_2_, 37 °C incubator in Dulbecco’s modified Eagle’s medium (DMEM including L-glutamine; SIGMA) supplemented with 10% fetal bovine serum and 100U penicillin/streptomycin.

### Western blotting

Cells were harvested with a rubber policeman, washed three times with PBS and resuspended in the lysis buffer (10 mM Tris, pH 7.4, 150 mM NaCl, 5 mM EDTA, 1% Triton X-100 and 1% NP-40). Proteins were fractionated by electrophoresis through 4–12% or 10% NuPAGE gel (Life Technologies) and then transferred to a nitrocellulose membrane (BIO-RAD). Membranes were incubated in the blotting solution for 2 h at room temperature, and then with a primary antibody at 4 °C overnight. Membranes were washed three times with PBS and incubated with a species-specific secondary antibody conjugated with horseradish peroxidase. Protein bands were visualized with a Western Lightning® ECL pro (PerkinElmer) in accordance with the manufacturer’s instructions.

### Immunofluorescence

Cells spread on a chamber slide (Watson) were fixed in 4% paraformaldehyde/phosphate buffered saline (PFA/PBS) solution (pH 7.4) for 30 min at 4 °C, and then permeated with 0.5% Triton X-100/PBS for 30 min at room temperature. Slides were incubated for preblocking with a blotting solution (10 mM Tris, pH 7.6, 150 mM NaCl, 0.5% skim milk, 0.2% bovine serum albumin and 0.01% Tween 20) for 1 h at room temperature, and then with a primary antibody at 4 °C overnight. After twice washing with PBS for 10 min, the cells were incubated with appropriate isotype-matched, AlexaFluor-conjugated secondary antibodies (Molecular Probes) for 1 h at 37 °C. Slides were mounted with Vectashield (Vector Laboratories) and viewed using a confocal microscope (Zeiss LSM780 or LSM880).

### Lysotracker & F-actin staining

Cells were incubated with LysoTracker Red DND-99 (Invitrogen/Molecular Probes, final concentration 100 nM) for 20 min at 37 °C. They were then fixed in 4% PFA/PBS solution for 30 min at 4 °C, washed twice with PBS at room temperature, and incubated with 100 nM Acti-stain ^TM^488 (Cytoskeleton) for 30 min at room temperature. Next the cells were washed twice with PBS, then incubated with DAPI for 10 min at room temperature. After two final washes with PBS, the slides were mounted with Vectashield (Vector Laboratories).

### EM

Culture cells on cover-slips were fixed in 3% glutaraldehyde in PBS, postfixed in 1% osmium tetroxide, dehydrated through graded ethanol steps and embedded in Epon 812. The cover-slips were peeled off and the sample was sectioned horizontally into ultrathin sections; the sections were then stained with uranyl acetate and lead citrate, and examined under a JEM 1400 (JEOL) electron microscope.

### si-RNA

Small interfering RNAs (siRNAs) against MSpin1 were used; the sequences were [5′-r(GCACUGGCACGAAAUCCUA)d(TT)-3′] and [5′- r(UAGGAUUUCGUGCCAGGC)d(TT)-5′] (SIGMA).

SiRNAs against the control were also used; these sequences were [5′-r(UAUAACAGUAUAUGAUAUC)d(TT)-3′] and [5′-r(GAUAUCAUAUACUGUUAU)d(TT)-3′] (JBiOS). Cells were transfected with 200 pmol siRNA using Fuge6 (Promega) according to the manufacturer’s protocol.

### Spin1-stably expressing cell line

A mouse Spin1-coding-sequence was isolated from whole mouse cDNA by using the primers:

MSpin1EcoRIATG (5′-ATACCGAATTCCATGGCCGGGTCCGACACGGC-3) and MSpin1BamHI (5-ATCGGATCCGTGATGAGCACGCTGGACACGGGGACT-3). The Spin1 PCR products were digested with *EcoR*I and *BamH*I. The enhanced green fluorescent protein (EGFP) vector was removed by *EcoR*I/BamHI digestion, and ligated with the PCR products.

To generate a cell line stably expressing Spin1, 573C10 cells were transfected by lipofection (Fugene6; Promega), in accordance with the manufacturer’s instructions. At day 3, G418 (GIBCO) was added to the cell culture at 600 μg/ml. Neomycin-resistant cells (GFPSpin1 expressing) were selected in culture for stable production of vector.

### LC3 turnover assay

Cells were cultured in DMEM medium containing 10% FBS or in EBSS (starvation condition) for 8 h in the presence or absence of protease inhibitors^36^, E64d (10 µg/ml) and pepstatin A (10 µg/ml). The cells were lysed, total proteins (15 µg per lane) were separated by SDS-PAGE, and endogenous LC3 in the lysates was recognized by immunoblotting with an anti-LC3 antibody.

### Statistical analysis

A Dunnet post-test was performed to detect statistically significant changes in the size of lysosomes or p62 during cultivation in normal medium, serum starvation medium or medium supplemented with L-leucine. A Dunnet post-test was performed to statistically evaluate differences in the amount of LC3 or p62 among the groups of cells cultured with different media. A Tukey post-test was performed to statistically evaluate differences in the signal intensity for LC3II/LC3, p-mTOR/mTOR or p62 among the groups of cells cultured with different media.

## Electronic supplementary material

Supplementary Information
