# Supplementary material for: L-leucine and SPNS1 coordinately ameliorate dysfunction of autophagy in mouse and human Niemann-Pick type C disease
Source: Sci Rep. 2017 Nov 21;7:15944. doi: 10.1038/s41598-017-15305-9 (PMC5698481; doi:10.1038/s41598-017-15305-9)
Supplement: Supplementary file 1 — Supplementary Information [file 41598_2017_15305_MOESM1_ESM.pdf]

Scientific Report

L-leucine and SPNS1 coordinately ameliorate dysfunction of autophagy in mouse and human Niemann-Pick type C disease

Hiroko Yanagisawa[*1], Tomohiro Ishii[2], Kentaro Endo[3], Emiko Kawakami[3], Keiko Akiyama[1], Kazuhiko Watabe[4], Masaaki Komatsu[5], Daisuke Yamamoto[6] and Yoshikatsu Eto[**1]

Key word: autophagy / NPC / L-leucine / Spin1/SPNS1 / LSD

Affiliation:

1. Advanced Clinical Research Center, Institute for Neurological Disorders, Kawasaki, Japan
2. Division of Pharmacology, Faculty of Pharmacy, Keio University, Tokyo, Japan
3. Center for Basic Technology Research, Tokyo Metropolitan Institute of Medical Science, Tokyo, Japan,
4. Department of Medical Technology, Faculty of Health Sciences, Kyorin University, Tokyo, Japan
5. Department of Biochemistry, Graduate School of Medical and Dental Sciences, Niigata University, Niigata, Japan
6. Division of Neurogenetics, Graduate School of Life Science, Tohoku University, Sendai, Japan

***Corresponding author**: Hiroko Yanagisawa, Tel: +81-44-322-0654 (Ext: 2785);
Fax: +81-44-966-0133; E-mail: hiro-yanagisawa@jikei.ac.jp

****Corresponding author**: Yoshikatsu Eto, Tel: +81-44-322-9991 (Ext: 3229);
Fax: +81-44-966-0133; E-mail: yosh@sepia.ocn.ne.jp

Figure S1

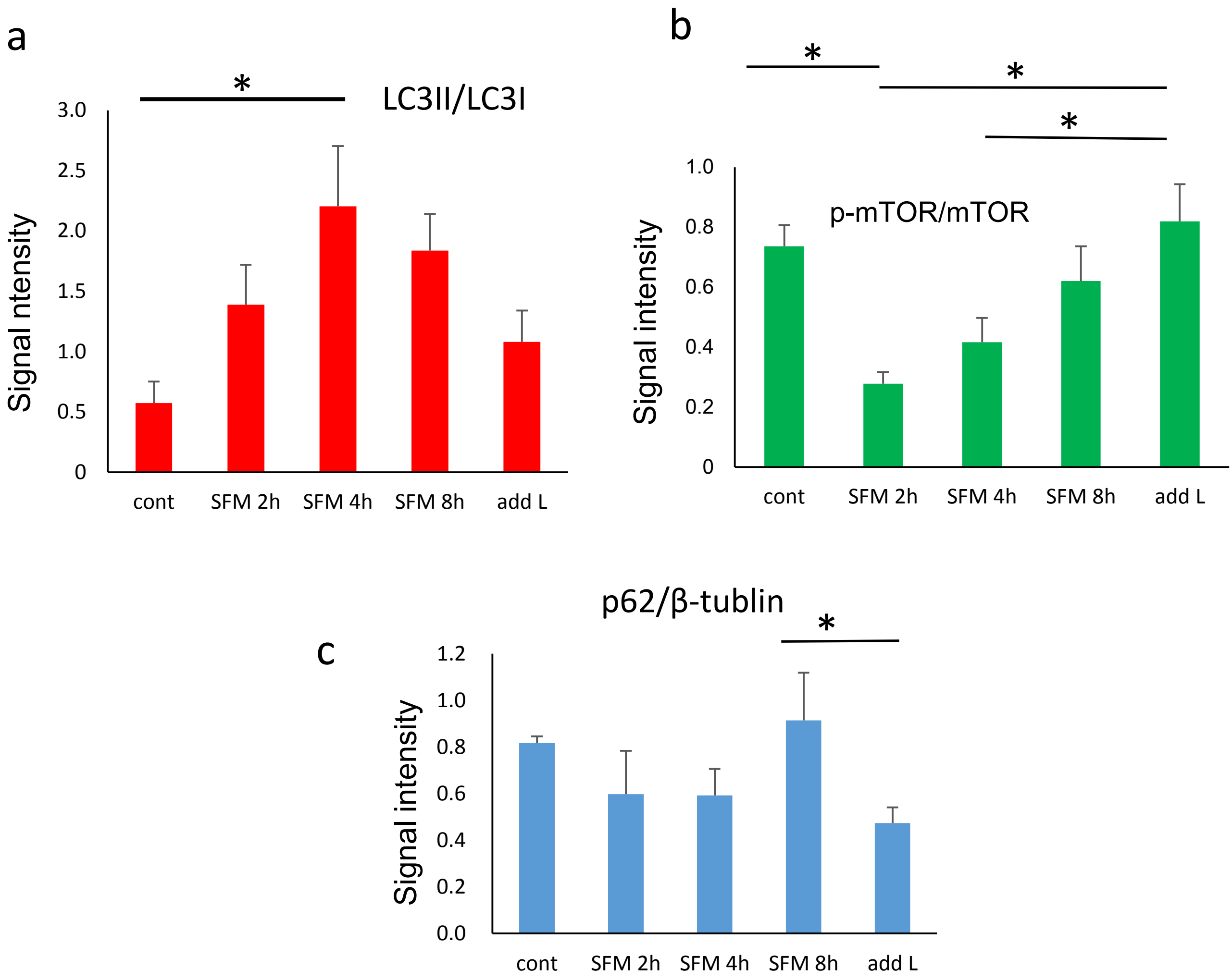

Serum free treatment induced autophagy in NPC(-/-) cells, reduction of phosphorylated mTOR and accumulation of p62 (*cf*. Fig.1O).

(a-c) The intensity of LC3II/LC3I (a), p-mTOR/mTOR (b) or p62 (c) signal was quantified with Fiji Image J (N=4). Statistical analysis was performed by the Tukey's test: * $P < 0.05$. The mean ± SEM are shown.

Figure S2

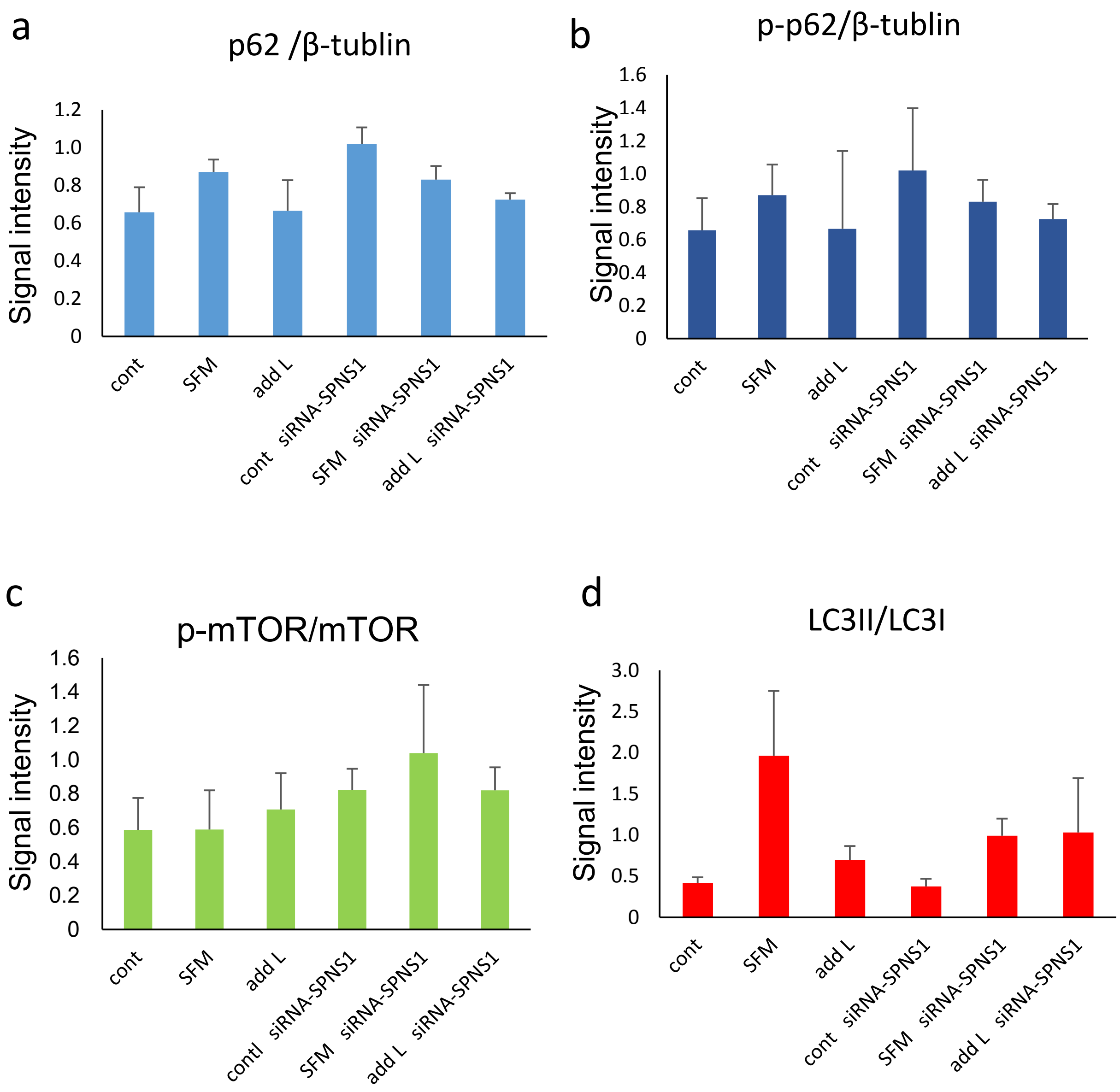

Effects of *siRNA-SPNS1* on the accumulation of p62 and phosphorylated p62 (*cf*. Fig. 2M).
(a-d) The intensity of p62 (a), p-p62 (b), p-mTOR/mTOR (c) or LC3II/LC3I (d) signal was quantified with Fiji Image J (N=3). The mean ± SEM are shown.

Figure S3

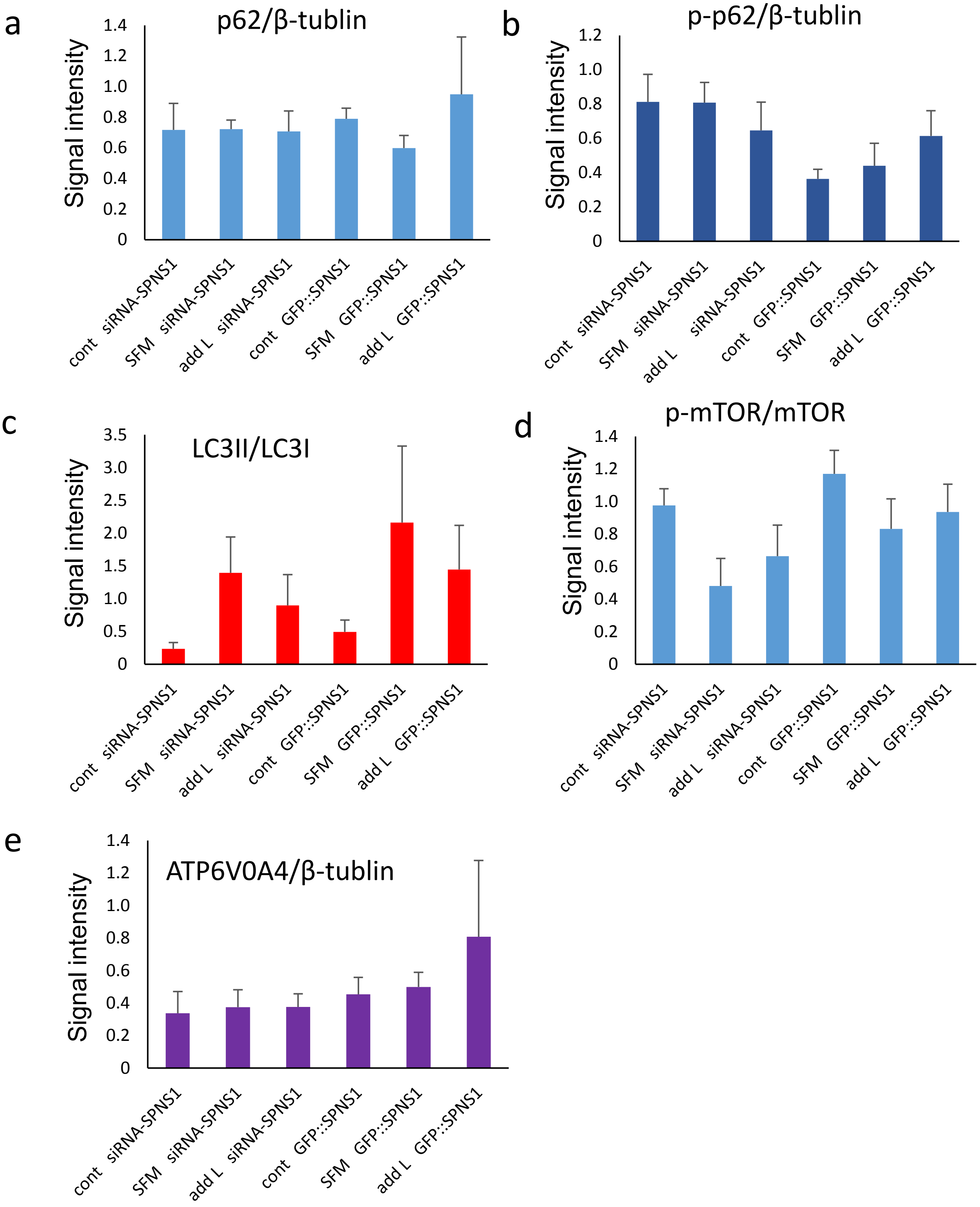

Effects of *SPNS1* overexpression on the expression of autophagy-related proteins (*cf*. Fig. 2N).

(a-e) The intensity of p62 (a), p-p62 (b), LC3II/LC3I (c), p-mTOR/mTOR (d) or ATP6V0A4 (e) signal was quantified with Fiji Image J (N=3). The mean ± SEM are shown.

Figure S4

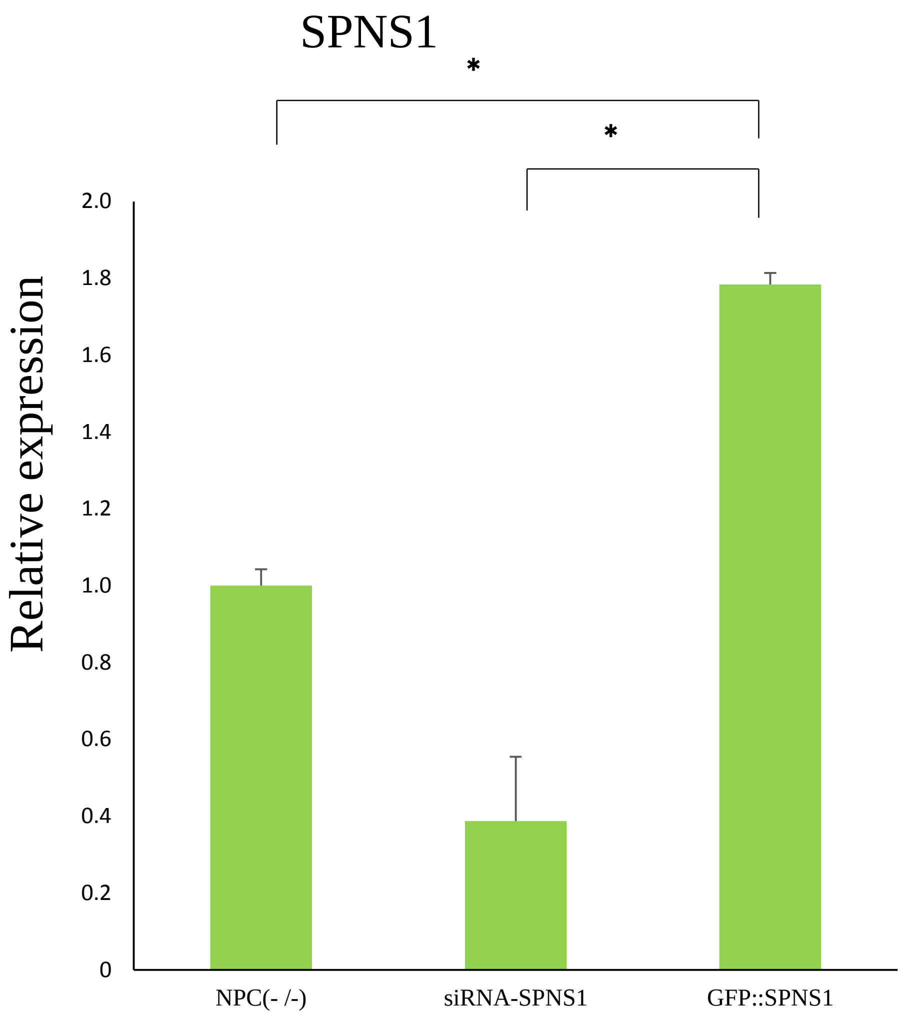

***SPNS1* expression was reduced by *siRNA-SPNS1*, and increased by overexpression of *GFP::SPNS1*.**

Samples in duplicate were subjected to PCR using the CFX96 Tough^{TM} Real-Time PCR Detection System (BIO-RAD). Relative expression was calculated based on the Ct values, as normalized to those of the housekeeping gene β-actin using the $2^{-\Delta\Delta Ct}$ method. Statistical analysis was performed by the Tukey's test: * $P < 0.05$. The mean ± SEM are shown. qPCR primers against human SPNS1were used; the sequences were [5'-CCTTCCTCAGCCAAGCAGAT-3] and [5'- CTATGAGGGTCGAATGGCCC-3'].

Figure S5

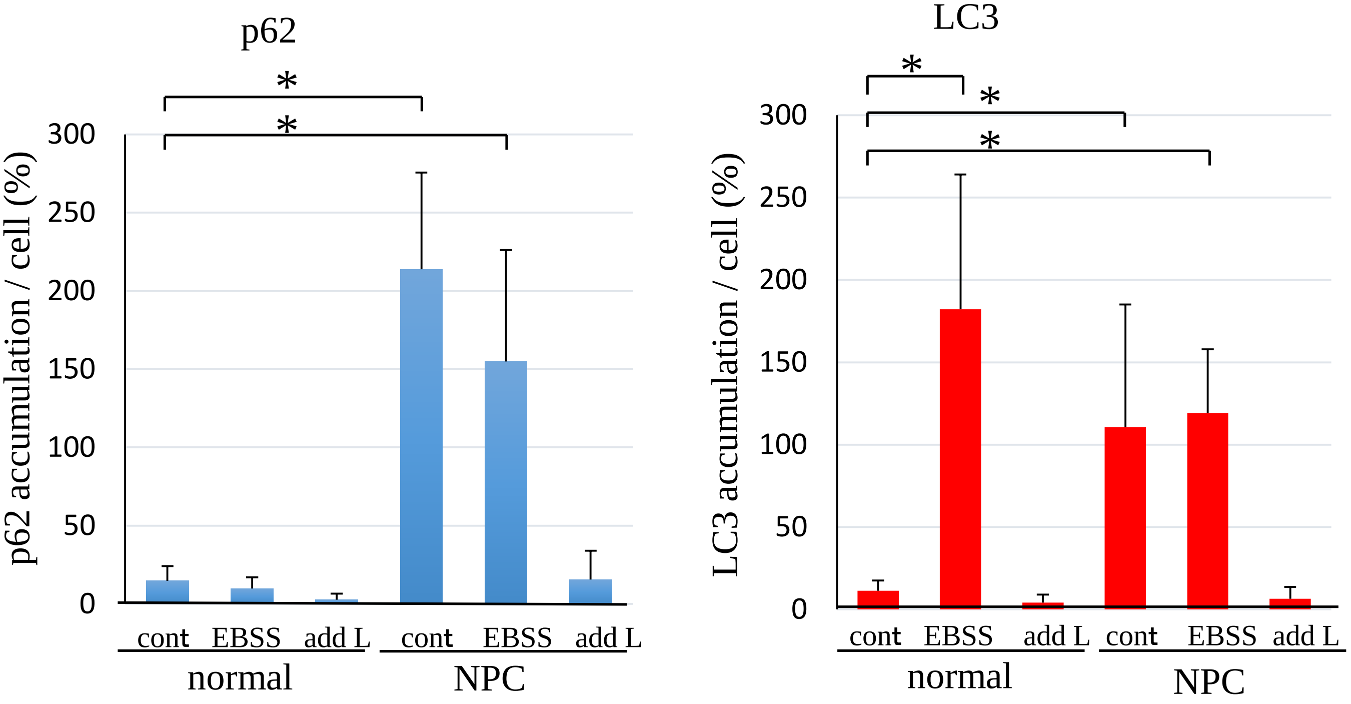

NPC-patient fibroblasts accumulated p62 and LC3 (*cf*. Fig. 3A).

Accumulattion of p62 and LC3 was analyzed quantitatively with an imaging software (Fiji Image J). Particles (small foci with a perimeter of less than 3.14 µm were not included) were counted in 10 ~ 13 image fields chosen at random for each condition (Dunnut's test: * p<0.05).

Figure S6

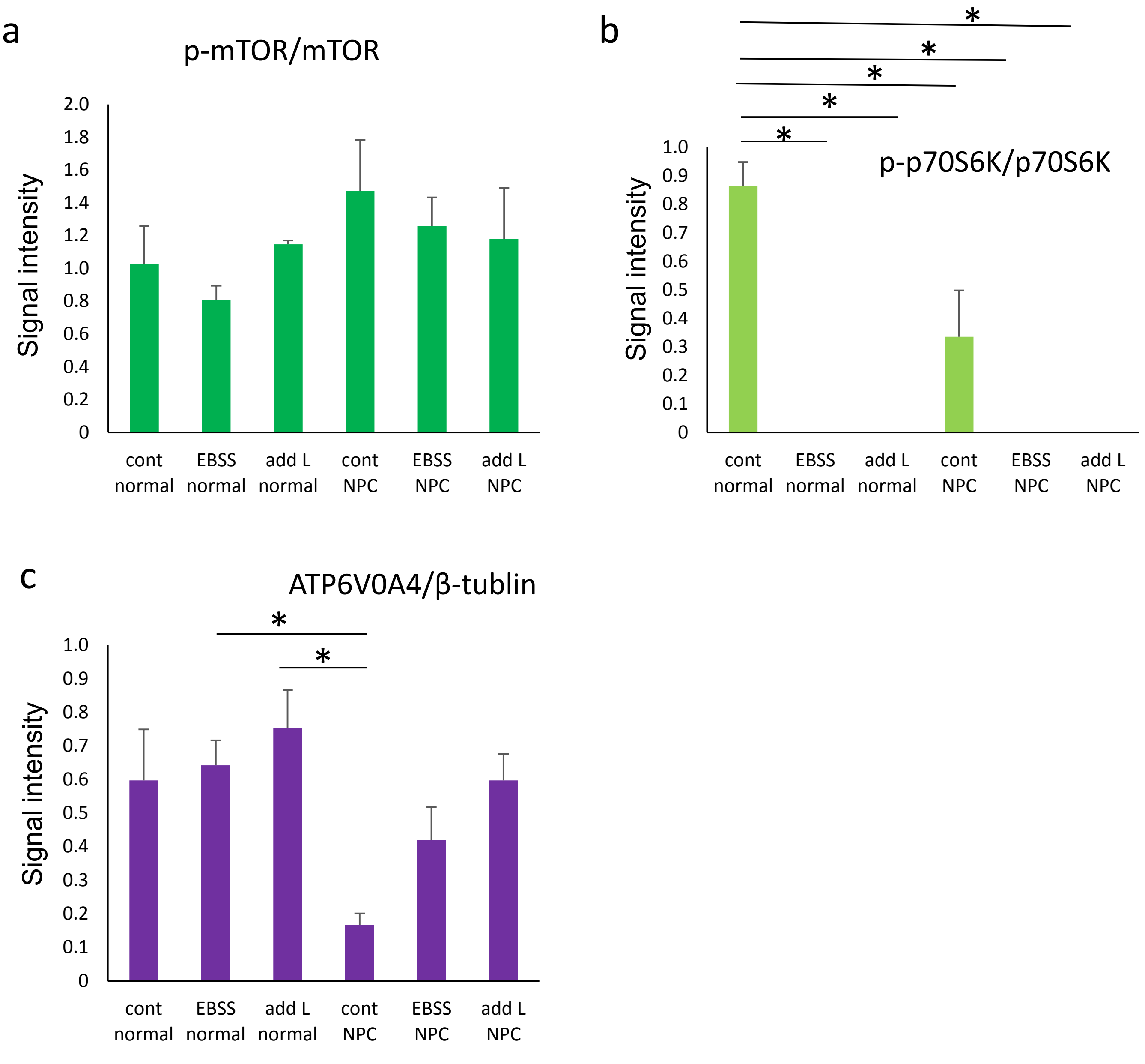

NPC-patient fibroblasts exhibited a reduction of p-p70S6k and ATP6V0A4 (*cf*. Fig. 3B).

(a-c) The intensity of p-mTOR/mTOR (a), p-p70S6K/p70S6K (b) or ATP6V0A4 (c) signal was quantified with Fiji Image J (N=3). Statistical analysis was performed by the Tukey's test: * P <0.05. The mean ± SEM are shown.

Figure S7

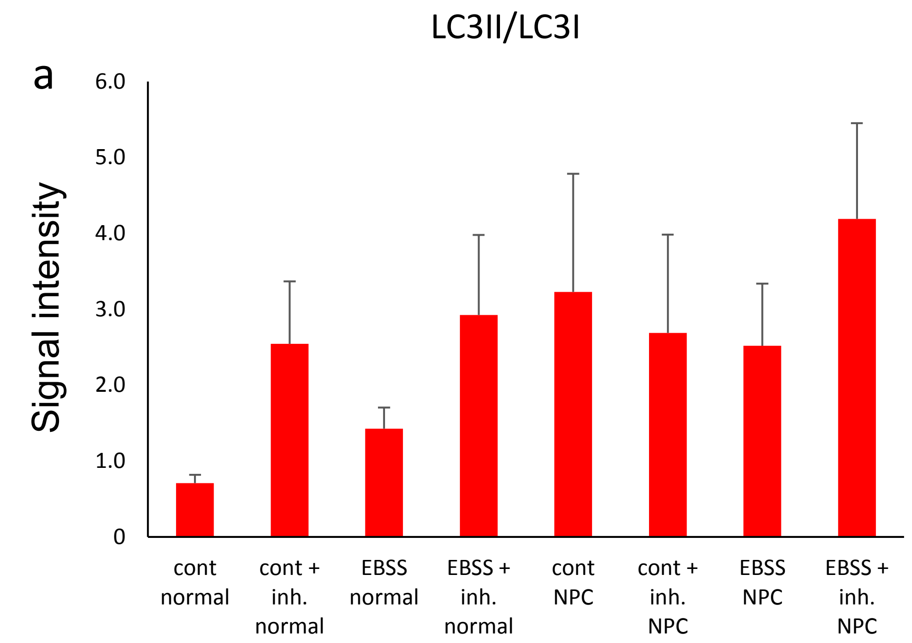

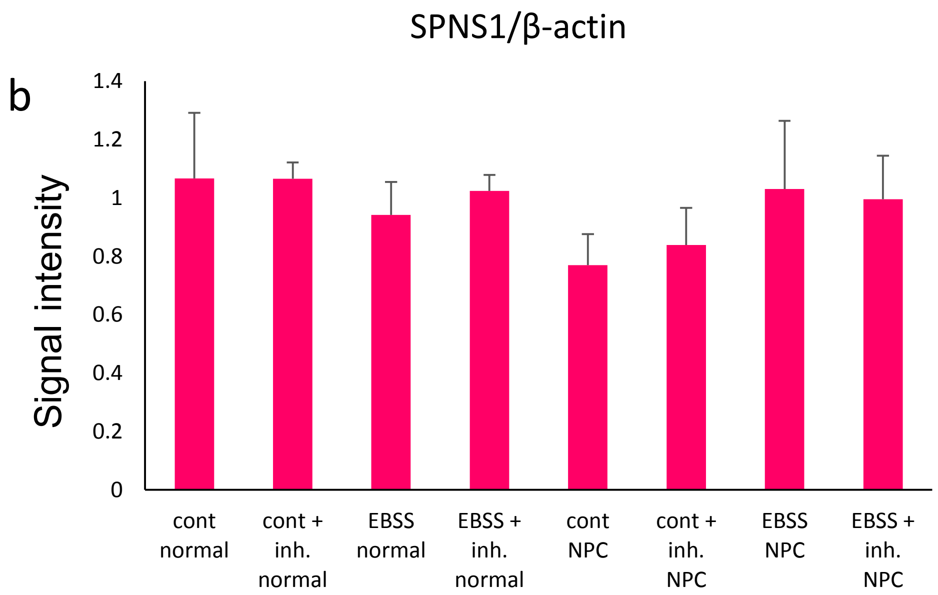

Expression of LC3II and *SPNS1* in normal and NPC fibroblasts
(*cf*. Fig. 3C).
(a,b) The intensity of LC3II/LC3I (a) or SPINS1 (b) signal was quantified with Fiji Image J (N=3). The mean ± SEM are shown.

Figure S8

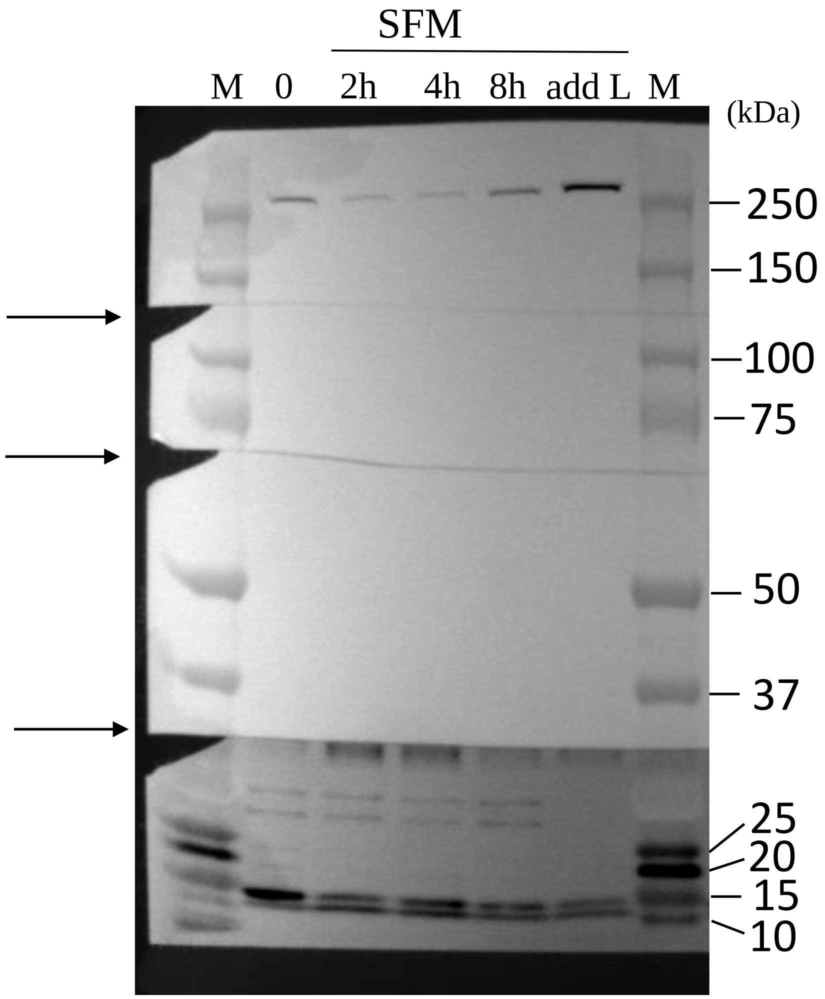

Fig. S8. A full length gel image containing the blots illustrated in Fig. 1O

Extracted proteins were electrophoresed on 4% to 12% polyacrylamide gels (NuPAGE Bis–Tris Mini Gels). Electrotransfered nitrocellulose membrane was cut into 4 areas as indicated with arrows. After cutting, the membranes were incubated with primary antibodies (the anti-phosphorylated mTOR antibody and the anti-LC3 antibody) and a secondary antibody (HRP-conjugated anti-rabbit IgG). Bands were visualized using the Western Lightning ECL pro (PerkinElmer) and the Ez-Capture II chemiluminescence imaging system (ATTO). The image was obtained with a 3 min exposure.

Figure S9

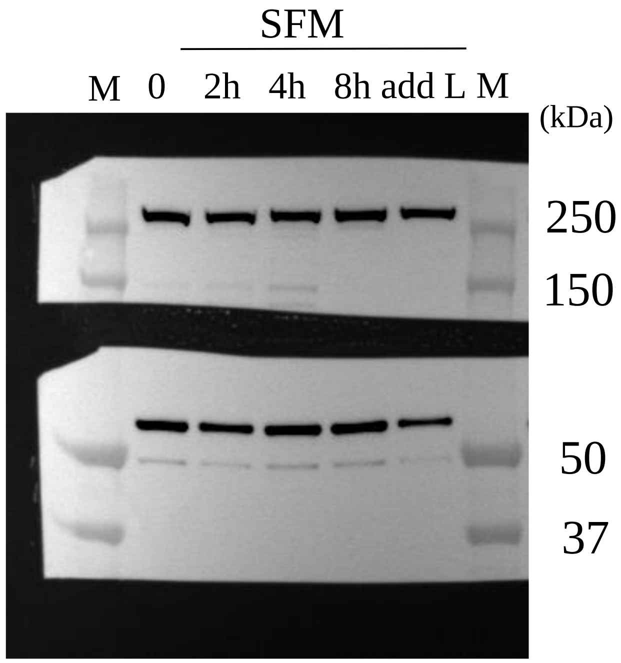

Fig. S9. Two slips of the membrane used in the experiment shown in Fig. S8 were re-probed with different antibodies to yield a blot shown in Fig. 1O.

The nitrocellulose membrane slips shown in Fig. S8 were incubated in Western Blot Stripping Buffer (Thermo Scientific Restore) at 37°C for 20 min to remove the first set of antibodies. The membranes were re-probed with primary antibodies (the anti-mTOR antibody and the anti-p62 antibody) and secondary antibodies (HRP-conjugated anti-rabbit IgG and HRP-conjugated anti-guinea pig IgG). Bands were visualized using the Western Lightning ECL pro (PerkinElmer) and the Ez-Capture II chemiluminescence imaging system (ATTO). The image was obtained with a 1 min exposure.

Figure S10

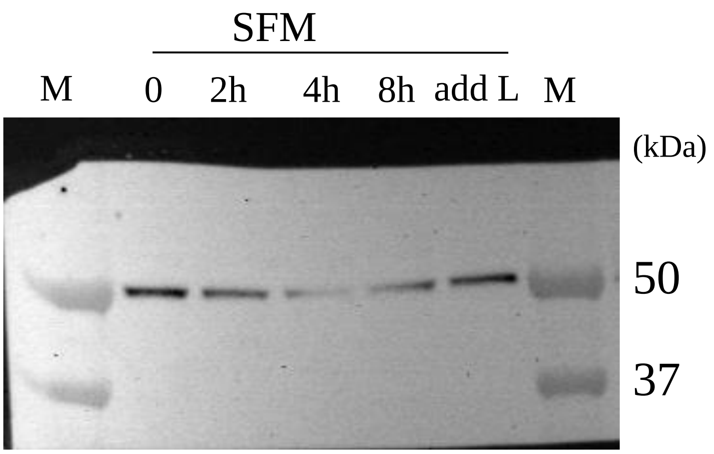

Fig. S10. A membrane slip used in the experiment shown in Fig. S9 was re-probed with a different antibody set.

The primary antibody was the anti-β tubulin antibody and the secondary antibody was HRP-conjugated anti-mouse IgG. The image was obtained with a 20 min exposure. Other details of the method are described in the Fig. S9 legend.

Figure S11

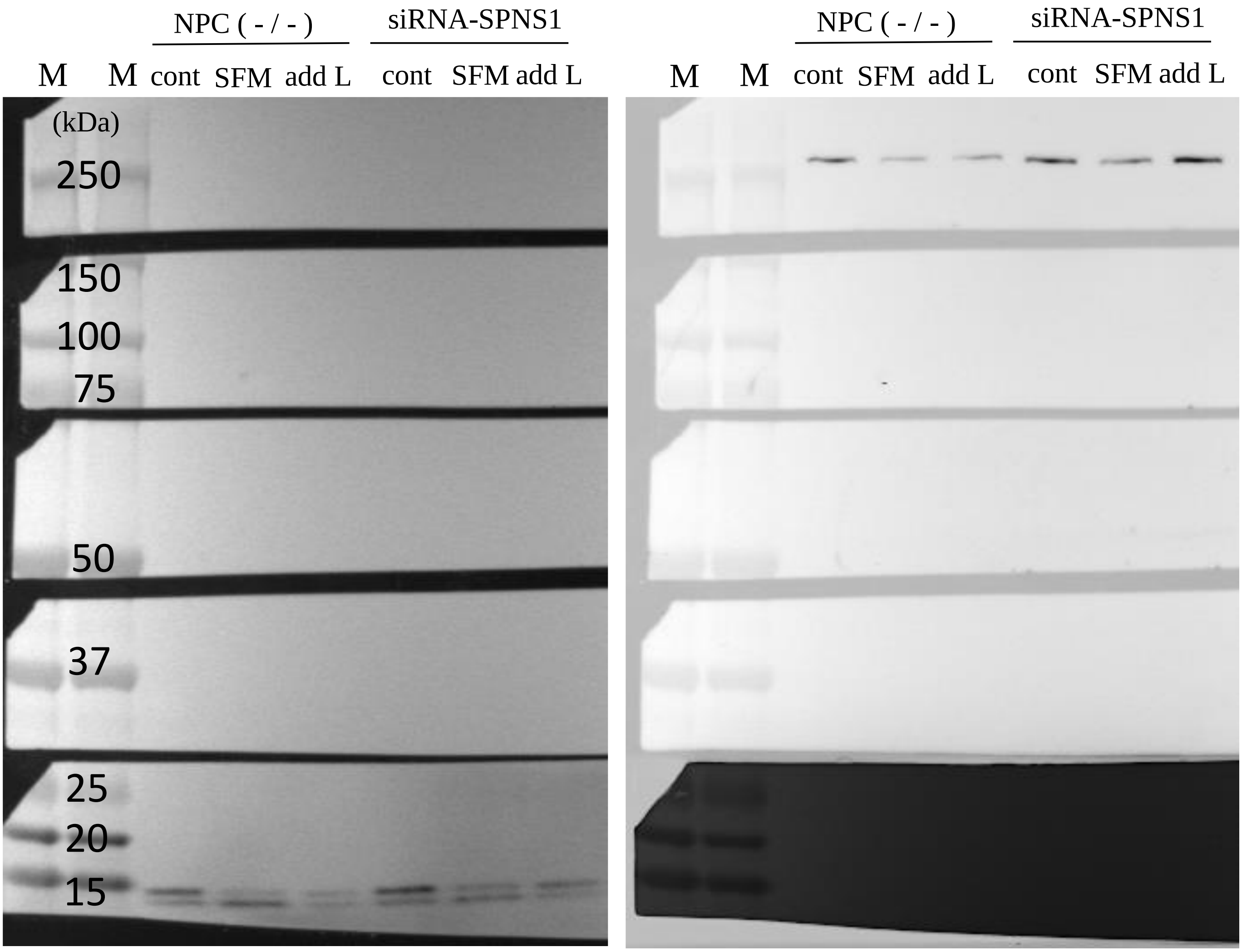

Fig. S11. A full length gel image containing the blots illustrated in Fig. 2M

The primary antibodies were the anti-LC3 antibody and the anti-phosphorylated mTOR antibody. The secondary antibody was HRP-conjugated anti-rabbit IgG. The left-hand side panel was obtained with a 1 min exposure. The right-hand side panel was obtained with a 10 min exposure. Other details of the method are described in the Fig. S8 legend.

Figure S12

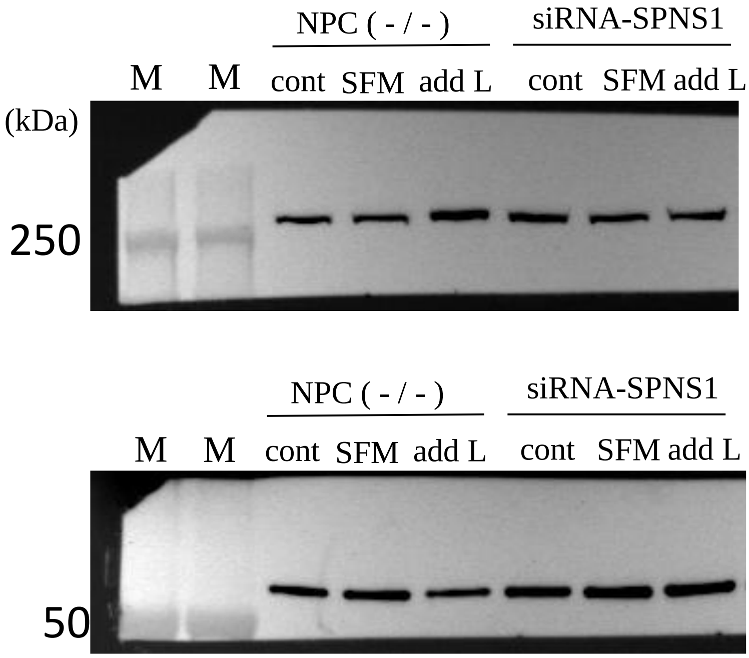

Fig. S12. Two slips of the membrane used in the experiment shown in Fig. S11 were re-probed with different antibodies to yield a blot shown in Fig. 2M.

The primary antibodies were the anti-mTOR antibody and the anti-p62 antibody. The secondary antibodies were HRP-conjugated anti-rabbit IgG and HRP-conjugated anti-guinea pig IgG. The upper panel was obtained with a 10 min exposure. The lower panel was obtained with a 20 min exposure. Other details of the method are described in the Fig. S9 legend.

Figure S13

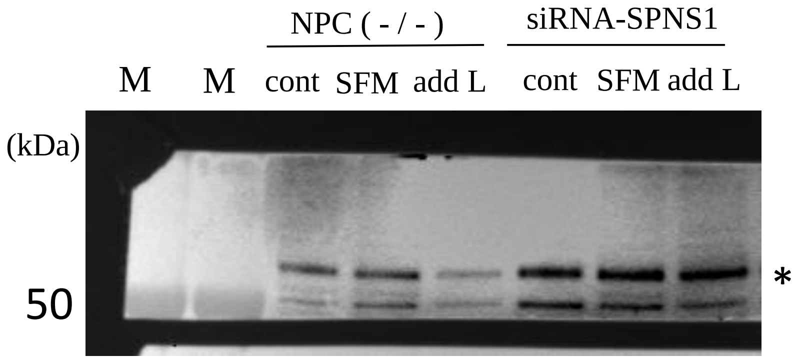

Fig. S13. A membrane slip used in the experiment shown in Fig. S12 was re-probed with a different antibody set.

The primary antibody was the anti-phosphorylated p62 antibody. The secondary antibody was HRP-conjugated anti-rabbit IgG. The image was obtained with a 5 min exposure. The band corresponding to phosphorylated p62 is indicated with an asterisk. Other details of the method are described in the Fig. S9 legend.

Figure S14

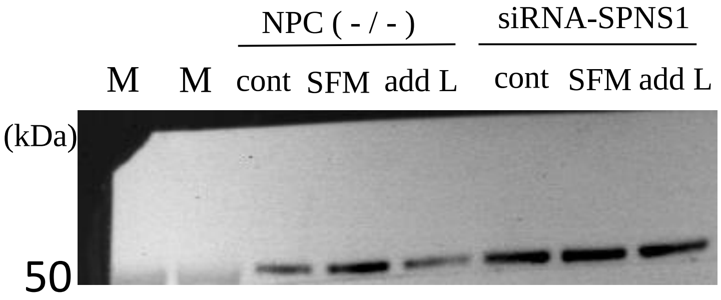

Fig. S14. A membrane slip used in the experiment shown in Fig. S13 was re-probed with a different antibody set.

The primary antibody was the anti-β tubulin antibody and the secondary antibody was HRP-conjugated anti-mouse IgG. The image was obtained with a 20 min exposure. Other details of the method are described in the Fig. S9 legend.

Figure S15

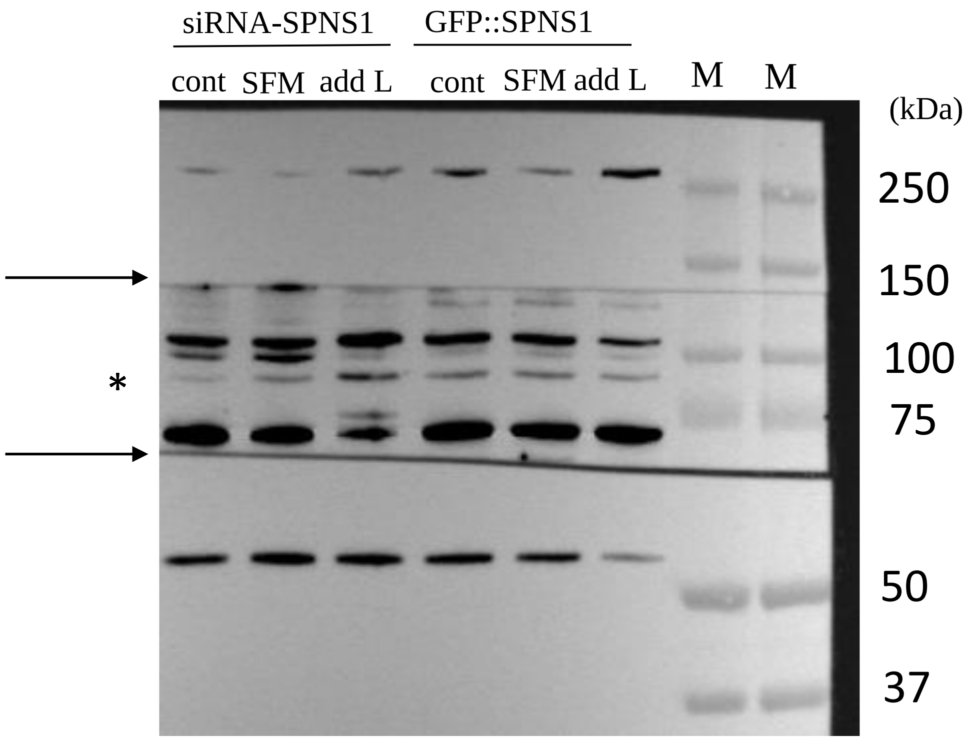

Fig. S15. A gel image containing the blots illustrated in Fig. 2N

Electrotransfered nitrocellulose membrane was cut into 3 areas as indicated with arrows. After cutting, the membranes were incubated with primary antibodies (the anti-phosphorylated mTOR antibody, the anti-ATP6V0A4 antibody and the anti-p62 antibody) and secondary antibodies (HRP-conjugated anti-rabbit IgG and HRP-conjugated anti-guinea pig IgG). The image was obtained with a 3 min exposure. The band corresponding to ATP6V0A4 is indicated with an asterisk.

Figure S16

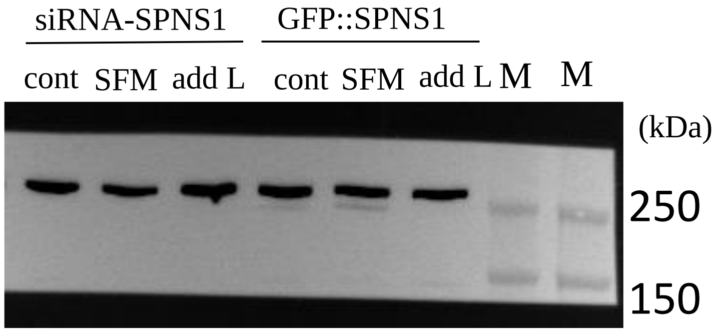

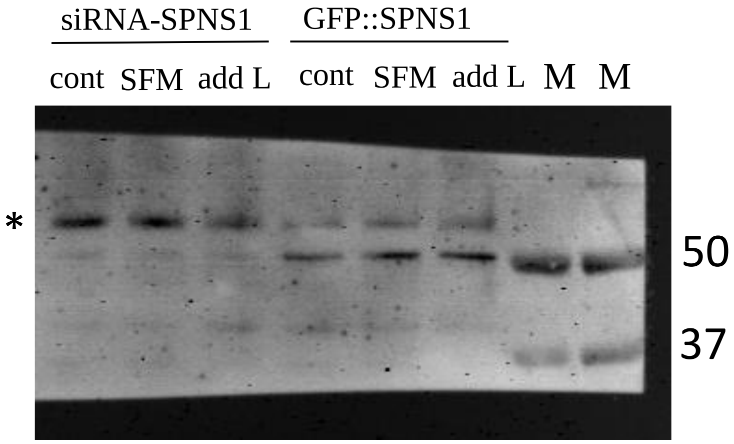

Fig. S16. Two slips of the membrane used in the experiment shown in Fig. S15 were re-probed with different antibodies to yield a blot shown in Fig. 2N.

Two slips of the membrane were re-probed with primary antibodies (the anti-mTOR antibody and the anti-phosphorylated p62 antibody) and a secondary antibody (HRP-conjugated anti-rabbit IgG). The upper panel was obtained with a 3 min exposure. The lower panel was obtained with a 1 h exposure. The band corresponding to phosphorylated p62 is indicated with an asterisk. Other details of the method are described in the Fig. S9 legend.

Figure S17

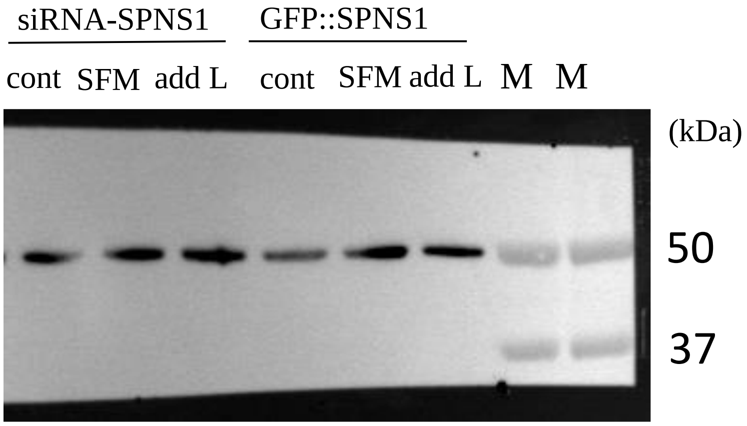

Fig. S17. A membrane slip used in the experiment shown in Fig. S16 was re-probed with a different antibody set.

The primary antibody was the anti-β tubulin antibody and the secondary antibody was HRP-conjugated anti-mouse IgG. The image was obtained with a 5 min exposure. Other details of the method are described in the Fig. S9 legend.

Figure S18

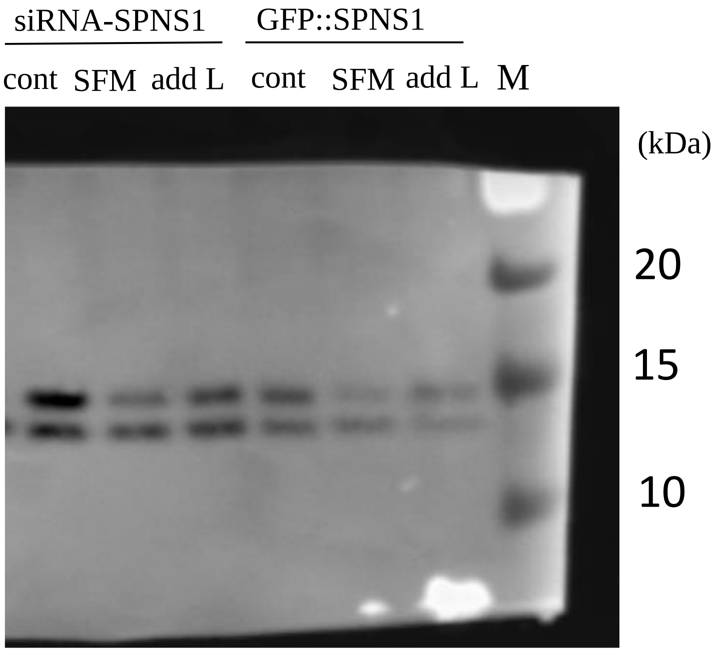

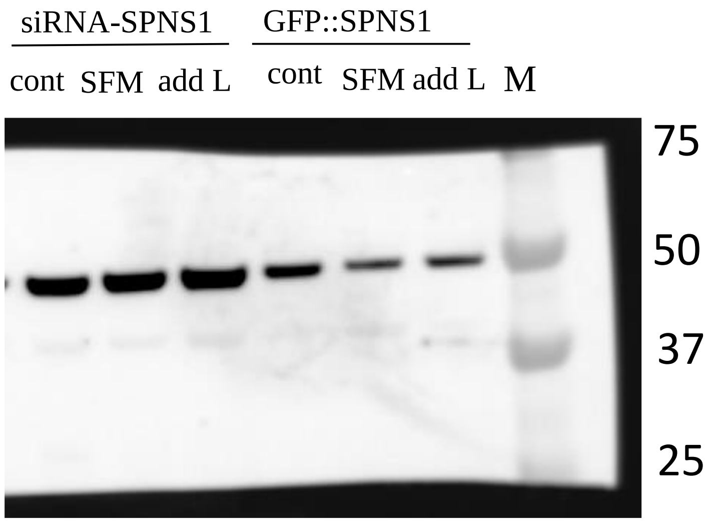

Fig. S18. A gel image containing the blots illustrated in Fig. 2N

Cell extracts were split into two fractions, one of which was used for experiment shown in Figure S15 and the other for this experiment. The extracts were subjected to electrophoresis in 10% NuPAGE Bis–Tris Mini Gels (Thermo Fisher). Electrotransfered nitrocellulose membrane (Bio-Rad) was cut into 2 areas. After cutting, the membranes were incubated with primary antibodies (anti-LC3 antibody and anti-β tubulin antibody) and secondary antibodies (HRP-conjugated anti-rabbit IgG and HRP-conjugated anti-mouse IgG). The upper panel was obtained with a 10 min exposure. The lower panel was obtained with a 20 min exposure.

Figure S19

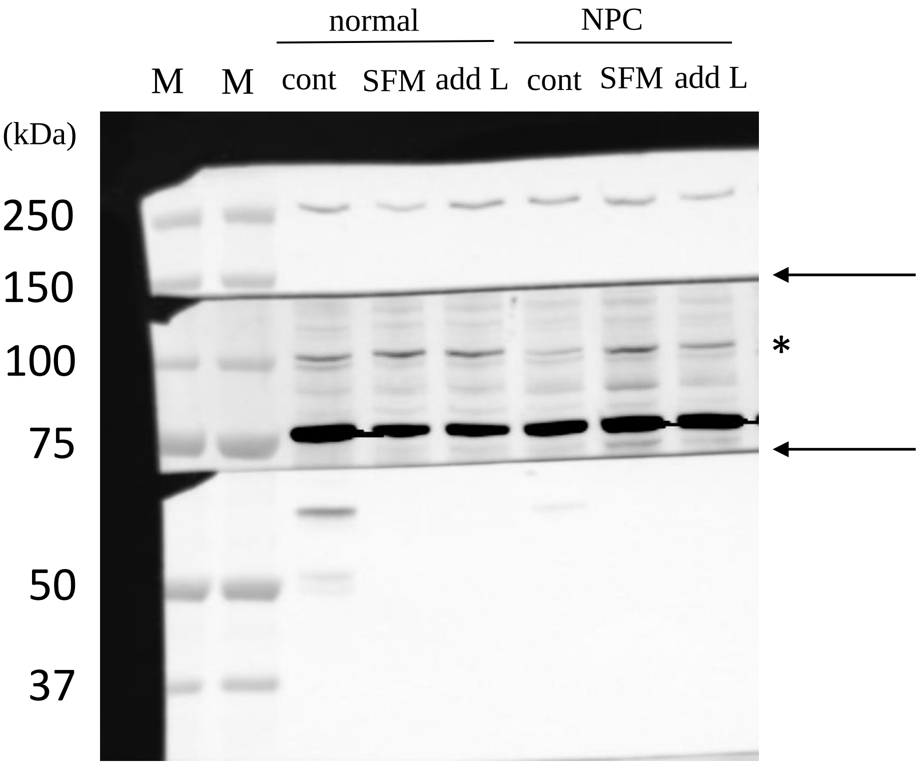

Fig. S19. A gel image containing the blots illustrated in Fig. 3B

Electrotransfered nitrocellulose membrane (Bio-Rad) was cut into 3 areas as indicated with arrows. After cutting, the membranes were incubated with primary antibodies (anti-phosphorylated mTOR antibody, anti-ATP6V0A4 antibody and anti-phosphorylated p70S6K antibody) and a secondary antibody (HRP-conjugated anti-rabbit IgG). The image was obtained with a 3 min exposure. The band corresponding to ATP6V0A4 is indicated with an asterisk.

Figure S20

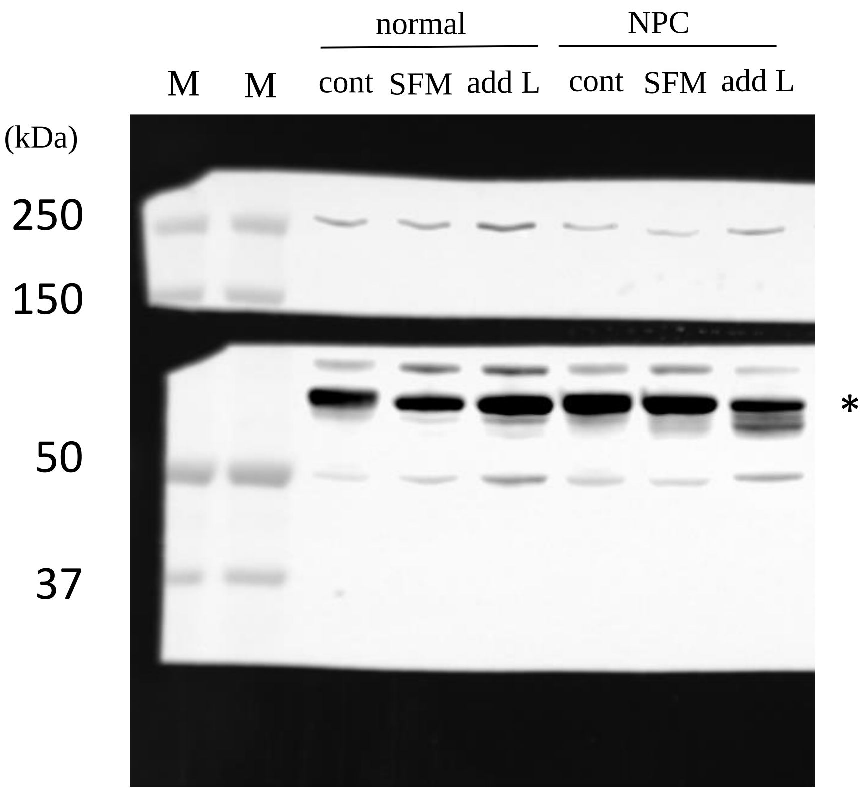

Fig. S20. Two slips of the membrane used in the experiment shown in Fig. S19 were re-probed with different antibodies to yield a blot shown in Fig. 3B.

The nitrocellulose membrane slips shown in Fig. S19 were incubated in Western Blot Stripping Buffer (Thermo Scientific Restore) at 37°C for 20 min to remove the first set of antibodies. The primary antibodies were the anti-mTOR antibody and the anti-p70S6K antibody. The secondary antibody was HRP-conjugated anti-rabbit IgG. The image was obtained with a 2 min exposure. The band corresponding to p70S6K is indicated with an asterisk.

Figure S21

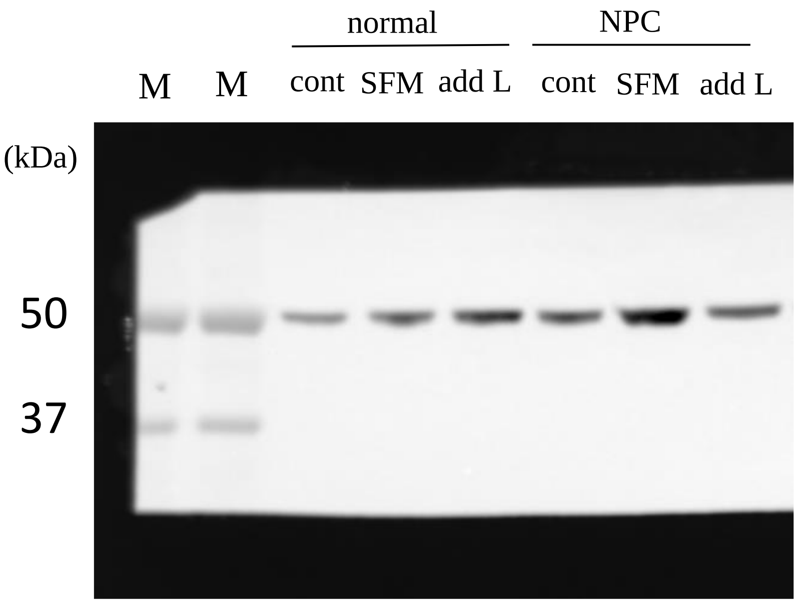

Fig. S21. A membrane slip used in the experiment shown in Fig. S20 was re-probed with a different antibody set.

The primary antibody was the anti-β tubulin antibody and the secondary antibody was HRP-conjugated anti-mouse IgG. The image was obtained with a 10 min exposure. Other details of the method are described in the Fig. S20 legend.

Figure S22

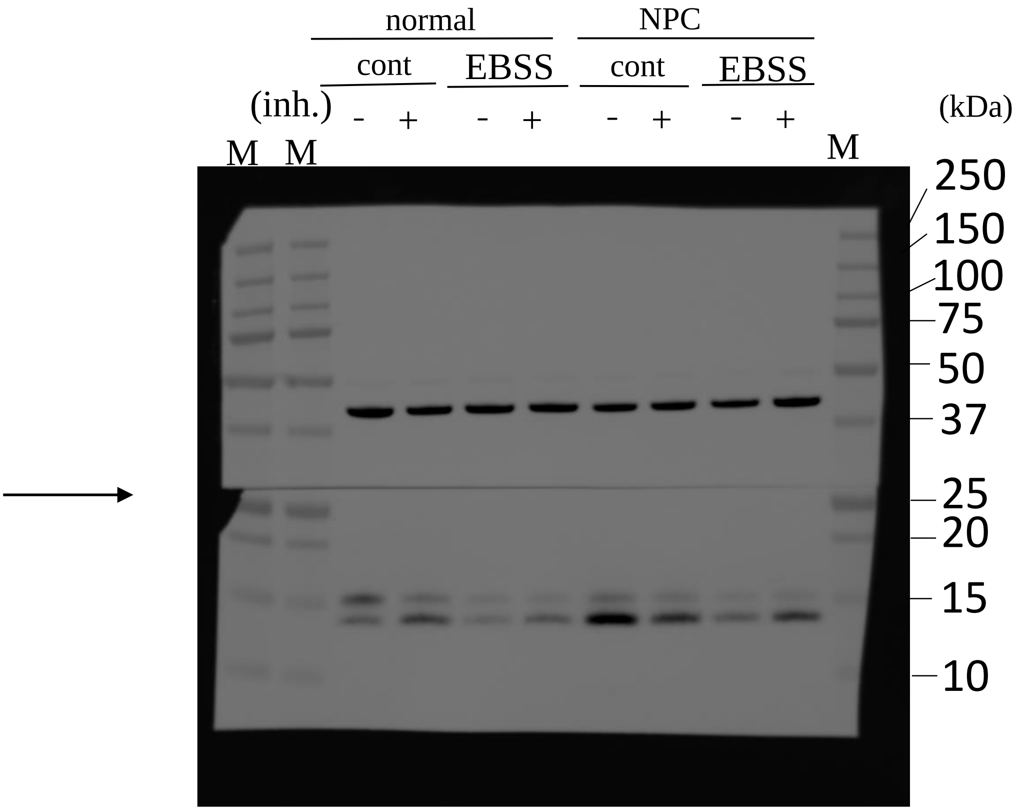

Fig. S22. A full length gel image containing the blots illustrated in Fig. 3C

Proteins were separated by electrophoresis in 10% NuPAGE Bis–Tris Mini Gels (Thermo Fisher). Electrotransfered nitrocellulose membrane (Bio-Rad) was cut into 2 areas as indicated with an arrow. After cutting, the membranes were incubated with primary antibodies (anti-β tubulin and anti-LC3 antibody) and secondary antibodies (HRP-conjugated anti-mouse IgG and HRP-conjugated anti-rabbit IgG). The image was obtained with a 1 min exposure.

Figure S23

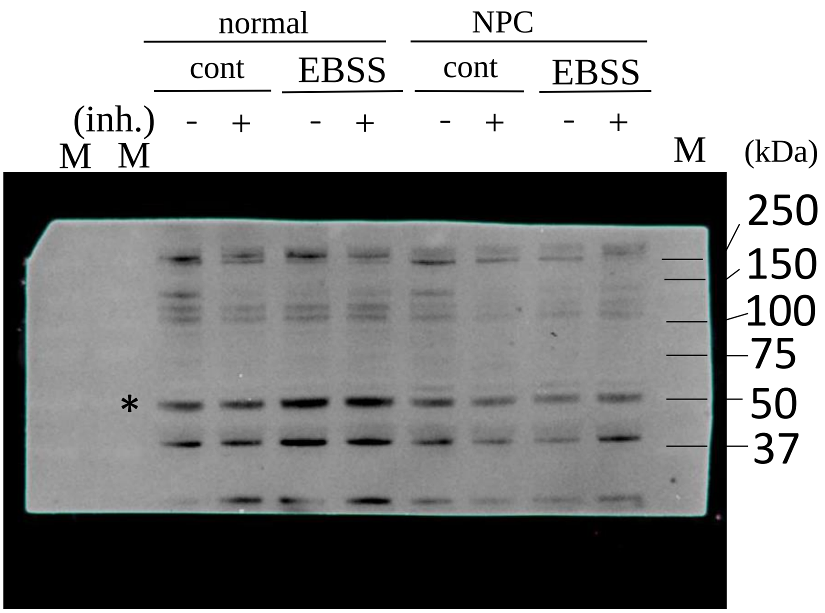

Fig. S23. A membrane slip used in the experiment shown in Fig. S22 was re-probed with a different antibody set.

The primary antibody was the anti-SPNS1 antibody and the secondary antibody was HRP-conjugated anti-rabbit IgG. The image was obtained with a 10 min exposure. The band corresponding to SPNS1 is indicated with an asterisk.
